# A Retrospective Trail Investigating Temozolomide Neoadjuvant Chemotherapy Combined with Radiotherapy in Low-Grade Pituitary Tumors

**DOI:** 10.1155/2022/4618664

**Published:** 2022-03-25

**Authors:** Jie Cui, Jianbo Shen, Xiaohong Ru, Zhihua Tian, Zhibin Duan, Guiping Chen, Min Li

**Affiliations:** Department of Neurosurgery, Jincheng People's Hospital, Jincheng, 048000 Shanxi Province, China

## Abstract

**Objective:**

To study and analyze the clinical application of temozolomide (TMZ) combined with radiotherapy in the treatment of low-grade pituitary tumors.

**Methods:**

A retrospective trail was conducted among 67 patients with low-grade pituitary tumors who were treated in our hospital from March 2018 to June 2020. According to different treatment methods, they were assigned into a combined group (37 cases, temozolomide capsules and radiotherapy) and a control group (30 cases, radiotherapy). The changes of serum prolactin (PRL), insulin-like growth factor-1 (IGF-1), GH levels, thyroid-stimulating hormone (TSH), serum free thyroxine (FT4), and adrenocorticotropic hormone (ACTH) were compared.

**Results:**

The chi-square test reports a significantly higher total effective rate in the combined group vs. control group (91.89% vs. 70.00%). Significant reductions in serum levels of PRL, IGF-1, and GH were observed in both groups after treatment, whereas the combined group treated with radiotherapy and TMZ resulted in significantly lower levels compared with the control group (*p* < 0.05). After treatment, TSH decreased, and FT4 and ACTH increased in both groups, and the treatment with radiotherapy and TMZ in the combined group led to a significantly greater amplitude of variation (*p* < 0.05).

**Conclusion:**

The combination of temozolomide and radiotherapy might be a promising technique for the treatment of pituitary tumors, thereby meriting promotion.

## 1. Introduction

Pituitary tumor is a common neuroendocrine benign intracranial tumor, approximately constituting 10-15% of intracranial tumors [1, 2]. Its main clinical manifestations are oppressive symptoms such as vision loss, diplopia, headache, and a series of endocrine abnormalities such as acromegaly, lactation, amenorrhea, and central obesity [3]. Albeit benign, most pituitary tumors aggressively grow, often invade the bone, dura mater, and other vital brain tissue structures, which challenges medical staff in seeking an effective therapy. Also, the properties of invasiveness raise the difficulty of surgical resection of the tumor and lead to a high recurrence rate after surgery. Worse yet is pituitary cancer may even metastasize to parts such as the spinal cord [4]. Despite that multiple techniques mainly surgical treatment of different approaches have been applied for pituitary tumor, their robustness is moderated by the somber outcomes and high recurrence rate [5]. Given the overall null clinical effects, there is a need to add drug therapy to counteract these adverse outcomes. Bromocriptine is a dopamine receptor agonist and acts by inhibiting the abnormal secretion of pituitary tumor hormones and diminishing tumor volume. The clinical efficacy of bromocriptine in the treatment of pituitary tumors has been verified and affirmed, yet with which tumor cannot be completely cured. As a result, an alternative temozolomide (TMZ) emerges in the treatment of pituitary tumors. TMZ is a new type of oral second-generation alkylating agent, an imidazole tetrazine derivative [6]. The main mechanism is through the alkylation of the 06 and N7 positions of guanine in DNA to induce cell apoptosis, thereby inhibiting pituitary tumors [7, 8]. TMZ, a cell cycle nonspecific drug with broad-spectrum antitumor activity, acts on the nucleic acid, protein, and peptide nucleophilic regions of tumor cells and various stages of tumor cell division [9]. This study was designed to explore the therapeutic effect of TMZ combined with chemotherapy in the treatment of pituitary tumors, with an aim to provide an experimental basis for application and clinical treatment with TMZ.

## 2. Study Design and Participants

### 2.1. Clinical Data

A retrospective trail was conducted among 67 patients with low-grade pituitary tumors who were treated in our hospital from March 2018 to June 2020. According to different treatment methods, they were assigned into a combined group of 37 cases and a control group of 30 cases. Inclusion criteria are as follows: (1) All patients meet the diagnostic criteria of pituitary tumor (comprehensively considering the patients' past medical history, symptoms, signs, auxiliary examination and postoperative pathology, etc. including cranial CT and MRI, requiring puncture biopsy or pathological examination after surgical resection), and are determined as patients with pituitary tumor without surgical contraindications; (2) imaging examination showed sellar space occupying; (3) patients with clinical symptoms of abnormal hormone metabolism; and (4) with nausea, vomiting, abnormal vision, headache, increased intracranial pressure, and other manifestations of compression of the surrounding pituitary gland. Exclusion criteria are as follows: (1) Patients with other types of diseases that cause hormonal abnormalities; (2) patients with liver and kidney dysfunction, blood system abnormalities, and pregnancy; (3) patients with immune system diseases; (4) patients taking hormone drugs within the past month; and (5) those allergic to the drugs used.

The combined group included 16 males and 21 females, aged 15-62 years, with an average age of 37.82 ± 4.07 years; the control group included 13 males and 17 females, aged 14-64 years, with an average age of 38.37 ± 4.24 years. Demographics and baseline characteristics were generally balanced between two groups (*p* > 0.05). All patients were informed about the trail and signed the informed consent form, and this study was in compliance with the protocol of the ethics committee of our hospital.

### 2.2. Treatment

The control group received radiotherapy, the size of the irradiation field was 4 cm × 4 cm~5 cm × 5 cm, the irradiation dose was 25.7~65.1 Gy (average irradiation dose 49.6 ± 5.12 Gy), and the number of irradiation was 15~35 times (average irradiation number 28.0 ± 1.1 times), each dose of 1.77 Gy (average dose of 1.70 ± 1.8 Gy per irradiation) for a total course of 30 to 62 days. On the basis of treatment in the control group, the combined group was given TMZ capsules (Jiangsu Tasly Diyi Pharmaceutical Co., Ltd., approval no. H20040637) 150 mg, 1 time/day orally, with 28-day course of treatment for a total of 3 courses.

### 2.3. Outcomes

Before and after treatment, 5 ml of fasting venous blood was collected for the determination of serum prolactin (PRL), insulin-like growth factor-1 (IGF-1), GH, serum thyroid stimulating hormone (TSH), serum free thyroxine (FT4), and adrenocorticotropic hormone (ACTH). Serum PRL was determined by electrochemiluminescence automatic immunoassay analyzer, serum IGF-1 was determined by enzyme-linked immunosorbent assay, and GH was determined by radioimmunoassay. Serums TSH, FT4, and ACTH were measured by electrochemiluminescence method to evaluate the recovery of pituitary function.

Efficacy assessment criteria are as follows: the establishment of efficacy assessment criteria was based on the volume of tumors, clinical symptoms, and hormone levels. Markedly effective: the volume of tumor was reduced by >70%, the mass effect was relieved, the hormone level basically returned to normal, the serum PRL level returned to normal, the clinical symptoms such as abnormal vision, headache, and increased intracranial pressure disappeared, and the pituitary function returned to normal. Effective: the tumor volume was reduced by 35%, the mass effect was weakened, the hormone level was effectively controlled, the serum PRL level was significantly reduced, the clinical symptoms such as abnormal vision, headache, and increased intracranial pressure were relieved, and the pituitary function was improved. Ineffective: before and after treatment, the tumor volume was reduced by 30%, the mass effect did not change or increased, hormone levels, pituitary function, serum PRL levels did not improve, and vision did not change or worsened. Total effective rate = [(number of effective cases + number of markedly effective cases)/total number] × 100%.

### 2.4. Statistical Analysis

All data analysis was performed with the SPSS 25.0 statistical software. Measurement data were verified via independent sample *t* test and described by (x ± s). Enumeration data were tested by the chi-square test and expressed as a percentage [*n* (%)]. The conventional *p* ≤ 0.05 was used to assess statistical significance.

## 3. Results

### 3.1. Baseline Data

Table 1 reveals that both groups of participants did not differ on demographic variables (*p* > 0.05).

### 3.2. Clinical Efficacy

The chi-square test reports a significantly higher total effective rate in combined group vs. control group (91.89% vs. 70.00%) (*p* < 0.05, Table 2).

### 3.3. Serum Levels

Significant reductions in serum levels of PRL, IGF-1, and GH were observed in both groups after treatment, whereas the combined group treated with radiotherapy and TMZ resulted in significantly lower levels compared with the control group (*p* < 0.05, Table 3).

### 3.4. TSH, FT4, and ACTH Levels

**Figure 1:**
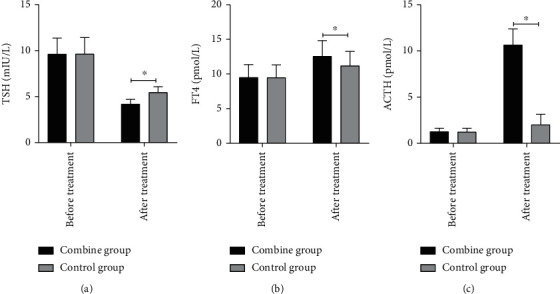
Comparison of the levels of TSH, FT4, and ACTH before and after treatment. Note: ^∗^ means <0.05.

**Figure 2:**
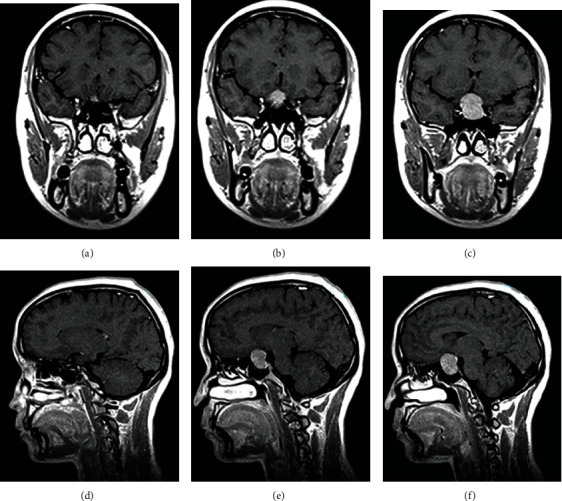
MRI finding of pituitary tumors.

After treatment, TSH decreased, and FT4 and ACTH increased in both groups, and the treatment with radiotherapy and TMZ in the combined group led to a significantly greater amplitude of variation (*p* < 0.05, Table 4 and Figures 1 and 2).

## 4. Discussion

Pituitary tumors are common primary tumors of the central nervous system [10], and local invasion often occurs in pituitary tumors, with manifestations as pituitary tumor invasion into the capsule and local extensive infiltration. With the advancements of medicine, the diagnostic criteria for invasive pituitary tumors are constantly proposed to be revised. However, until now, there remain no complete and universal diagnostic criteria, so the treatment methods are inconsistent. At present, the mainstays include surgical resection and radiation therapy [11]. However, it is difficult to completely remove with surgery alone due to the property of invasive growth of tumors, and the conjunction treatment plan of surgery-assisted radiation therapy and drug therapy has always been the mainstay.

Gamma knife [12], a part of radiosurgery, has been universally applied in the treatment of pituitary tumors, especially invasive pituitary tumors, given its small incision and less adverse reactions, and it can be used as a preferred option for certain patients [13, 14]. TMZ, as a second-generation alkylating agent, can induce DNA damage and inhibit DNA transcription at the 0-6 guanine bases [15]. Currently, it is dominantly used for the treatment of malignant glioma, malignant melanoma, and brain metastases and has achieved certain effect in the treatment of pituitary tumors [16]. This trail was specially designed to fill the gap of postoperative radiotherapy combined with TMZ in pituitary tumors. We firstly observed that the changes of PRL, IGF-1, and GH levels in the combined group and the control group decreased, and the degree of reduction in the combined group was greater. To our knowledge, VEGF is a powerful angiogenic factor in solid tumors [17]. In the present study, we found that VEGF in the cells of the combined group was significantly reduced, and the inhibiting function was more potent than that of the group with radiotherapy alone, suggesting a robust outcome of combination therapy. Consistently, Baldys et al. also found that the combined group has lower PRL and GH secretion than radiation group [18], but it remains vague whether the decreased PRL results from inhibition of VEGF secretion, and further study of its mechanism was needed. TSH, FT4, and ACTH serve as central indicators reflecting pituitary function in patients with pituitary tumors [19]. According to our results, TSH decreased, and FT4 and ACTH increased in both groups, and the treatment with radiotherapy and TMZ in the combined group led to a significantly greater amplitude of variation. To our best understanding, the pituitary dysfunction caused by pituitary tumors mainly manifests as thyroid and adrenal dysfunction. The above results demonstrated that TMZ combined with chemotherapy in the treatment of pituitary tumors can effectively inhibit the occurrence of thyroid and adrenal hyperfunction [20].

Taken together, TMA plus radiation might be a preferred option in the treatment of pituitary tumors, and this study provides an experimental basis for the future application of TMZ combined with radiation.

## Figures and Tables

**Table 1:** Baseline data.

	Combined group	Control group	*X* ^2^/*t*	*p*
Gender (male/female)	16/21	13/17	0.0	0.994
Age	15~62	14~64		
Mean age	37.82 ± 4.07	38.37 ± 4.24	0.529	0.599

**Table 2:** Comparison of clinical efficacy [*n*(%)].

Groups	*n*	Markedly effective	Effective	Ineffective	Total
Combined group	37	14	20	3	34 (91.89)
Control group	30	9	12	9	21 (70.00)
*X* ^2^					5.4
*p*					0.02

**Table 3:** Comparison of serum levels ($\bar{\text{x}}$±s).

Groups	*n*	PRL (*μ*g/L)		IGF-1 (ng/L)		GH (*μ*g/L)	
		Before treatment	After treatment	Before treatment	After treatment	Before treatment	After treatment
Combined group	37	131.59 ± 12.77	25.09 ± 5.24	0.48 ± 0.12	0.24 ± 0.15	16.31 ± 1.88	3.36 ± 0.85
Control group	30	131.42 ± 13.28	45.13 ± 14.21	0.49 ± 0.11	0.36 ± 0.17	16.18 ± 1.90	8.03 ± 1.07
*t*		0.053	7.949	0.352	3.067	0.28	19.916
*p*		0.958	<0.001	0.726	0.003	0.78	<0.001

**Table 4:** Comparison of the levels of TSH, FT4, and ACTH ($\bar{\text{x}}$±s).

Groups	n	TSH (mIU/L)		FT4 (pmol/L)		ACTH (pmol/L)	
		Before treatment	After treatment	Before treatment	After treatment	Before treatment	After treatment
Combined group	37	9.62 ± 1.74	4.18 ± 0.52	9.49 ± 1.87	12.56 ± 2.26	1.27 ± 0.38	10.66 ± 1.72
Control group	30	9.64 ± 1.78	5.44 ± 0.63	9.47 ± 1.83	11.18 ± 2.07	1.23 ± 0.40	2.01 ± 1.15
*t*		0.046	8.971	0.044	2.58	0.418	23.585
*p*		0.963	<0.001	0.965	0.012	0.677	<0.001

## Data Availability

The datasets used during the present study are available from the corresponding author upon reasonable request.
